# The mediating role of feelings of hopelessness and repetitive negative thinking in the relationship between perfectionism and depressive symptoms among medical students with suicidal ideation

**DOI:** 10.3389/fpsyt.2025.1560653

**Published:** 2025-04-28

**Authors:** Ali Hosseinzadeh Oskouei, Reyhaneh Sardarzehi, Mohammad Sadegh Zamani Zarchi, Seyed Alireza Tavallaei Zavareh, Jamal Shams, Amir Sam Kianimoghadam, Abbas Masjedi Arani

**Affiliations:** ^1^ Department of Clinical Psychology, School of Medicine, Shahid Beheshti University of Medical Science, Tehran, Iran; ^2^ Faculty of Psychology and Educational Sciences, Kharazmi University, Tehran, Iran; ^3^ Student Research Committee, School of Medicine, Shahid Beheshti University of Medical Sciences, Tehran, Iran; ^4^ Behavioral Science Research Center, Shahid Beheshti University of Medical Sciences, Tehran, Iran; ^5^ Department of Clinical Psychology, Taleghani Hospital Clinical Research Development Unit, Shahid Beheshti University of Medical Sciences, Tehran, Iran

**Keywords:** depression, hopelessness, medical students, negative thinking, perfectionism, suicidal ideation

## Abstract

**Background:**

Perfectionism and depressive symptoms are among psychological concerns in medical students with suicidal ideation. While studies suggest a link between perfectionism and depression, the role of mediating mechanisms remains unclear.

**Objective:**

This study aimed to examine the mediating role of hopelessness and repetitive negative thinking in the relationship between perfectionism and depressive symptoms among Iranian medical students.

**Methods:**

The research employed a descriptive-correlational design using structural equation modeling (SEM). The study population comprised all medical students in Tehran in 2024, from which 355 individuals were selected through purposive sampling. Data were collected using the Beck Scale for Suicidal Ideation (BSSI), Tehran Multidimensional Perfectionism Scale (TMPS), Beck Depression Inventory (BDI-II), Repetitive Negative Thinking Questionnaire (RNTQ), and the Beck Hopelessness Scale (BHS). The data were analyzed using structural equation modeling.

**Results:**

The results indicated that the proposed model demonstrated an excellent fit, and perfectionism was significantly and directly associated with depressive symptoms (p < 0.01). Additionally, perfectionism indirectly predicted depressive symptoms (p < 0.001) through hopelessness (β = 0.35) and repetitive negative thinking (β = 0.37). Together, perfectionism, hopelessness, and repetitive negative thinking accounted for 58% of the variance in depressive symptoms (R² = 0.58).

**Conclusions:**

These findings suggest that perfectionism contributes to an increase in depressive symptoms both directly and indirectly, by intensifying feelings of hopelessness and repetitive negative thinking.

## Introduction

Suicidal ideation (SI) is a wide-ranging term used to explain contemplations, preoccupations, and wishes of death and suicide (1). Medical students have been identified as a high-risk group for SI, with prevalence rate ranging from 1.8% to 53.6%. (2–4). Poor mental health conditions, including depression, are significant risk factors for SI in medical students (5). In fact, the rate of depression among medical students is higher than that found in the general population (6), and their symptoms tend to worsen as they progress through their medical training (7). Therefore, given the elevated rates of depression and the tendency for symptoms to worsen during medical training, clinical literature consistently emphasizes the importance of identifying potential vulnerabilities to depressive symptoms in this population (8). This understanding is crucial for formulating effective strategies for prevention, intervention, and treatment of these symptoms.

Perfectionism is one such vulnerability factor, and its role in the development of depressive symptoms has been the subject of extensive investigation over several decades (9). This concept can be divided into three dimensions: self-oriented, other-oriented, and socially prescribed perfectionism. Self-oriented perfectionism involves establishing high personal standards, while other-oriented perfectionism refers to imposing unrealistic expectations on others. In contrast, socially prescribed perfectionism occurs when individuals believe they are pressured by others to meet excessively high standards (10). An increasing body of evidence indicates that the vulnerabilities linked to depressive symptoms may be triggered only in the presence of certain moderating or mediating factors (8, 9). According to previous studies, negative consequences associated with perfectionism, such as anti-mattering (11), and lower levels of self-compassion (12), play a significant role in the development of these symptoms among student samples. However, two variables that have not been investigated in studies on perfectionism and depressive symptoms in this population are feelings of hopelessness and repetitive negative thinking (RNT). Therefore, this study aims to investigate the potential mediating roles of feelings of hopelessness and RNT in the relationship between perfectionism and depressive symptoms in medical students.

Feelings of hopelessness represent a pessimistic attitude or negative expectation about oneself and the future, contributing to a tendency to overestimate negative events while underestimating the probability of positive outcomes (13). This construct was chosen due to its established clinical significance, as several studies have shown that greater feelings of hopelessness are related to both an increase in SI (14) and other depressive symptoms (15). Strong evidence suggests that feelings of hopelessness, acting as a cognitive vulnerability factor, uniquely contributes to the development of depression (16). Furthermore, perfectionism contributes to increased feelings of hopelessness (17, 18). Additionally, consistent with the perfectionism social disconnection model (PSDM), Smith et al. (19) propose that socially prescribed perfectionism relates to depressive symptoms after five months, mediated by social hopelessness (19). While existing research has explored these connections broadly (9, 19), no studies have examined whether hopelessness mediates the perfectionism-depressive symptoms link specifically in medical students, particularly those with SI.

The other construct proposed to mediate the effect of perfectionism on depressive symptoms is RNT. Generally, RNT refers to a repetitive, intrusive, passive, and self-focused thinking about past events, future concerns, and one’s problems, in a way that is difficult to disengage from (20). The common types of RNT consist of worry and rumination. Both have been shown to be potential underlying factors contributing to depressive symptoms (21). The perfectionism cognition theory may also provide an explanation for the mediating role of RNT. It focuses on how various forms of RNT contribute to the perpetuation of a cycle involving perfectionism and psychopathology. This model suggests that perfectionists often experience various forms of cognitive perseveration, such as rumination over failures and mistakes, and social comparison. These cognitive perseverations can contribute to mental health disorders (22). ​Consequently, RNT is proposed as a mediator in the relationship between perfectionism and depressive symptoms. However, there is a notable gap in research, as no studies have examined RNT as a mediator between perfectionism and depressive symptoms in medical students experiencing SI, which this study aims to address.

### The current study

In summary, the current study hypothesizes whether perfectionism is related to depressive symptoms in medical students with SI, and whether RNT and feelings of hopelessness play a chain mediating role in this relationship. Specifically, our study is based on the following hypotheses:

Hypothesis 1: Perfectionism would be positively related to depressive symptoms in medical students with SI.

Hypothesis 2: Feelings of hopelessness would mediate the relationship between perfectionism and depressive symptoms in medical students with SI.

Hypothesis 3: RNT would mediate the relationship between perfectionism and depressive symptoms in medical students with SI.

Accordingly, this study explores the mediating effect of RNT and feelings of hopelessness in the relationship between perfectionism and depressive symptoms, providing valuable insights for preventive interventions to address perfectionism and promote mental health among medical students with SI.

## Methods

This study employed a descriptive-correlational design using structural equation modeling (SEM). The target population consisted of undergraduate medical students in Tehran with suicidal ideation (SI) in 2024. On the basis of Klein’s point of view, 355 students were selected as a sample using the purposive sampling method (23). Eligible participants were those who met these inclusion criteria: being medical student at the universities of Tehran, presence of suicidal thoughts within the last week as well as a score from 1 to 5 on the first five questions of the BSSI used for screening, no history of acute mental and physical disorders, based on the self-reports no history of psychiatrist visit and receiving psychiatric medication, and not experiencing a life crisis during the last year, such as the death of loved ones. The exclusion criteria included incomplete questionnaires and single-variable and multi-variable outlier data. To collect data, the authors first visited medical universities in Tehran, explained the study objectives to the students, and invited those interested to participate by providing their phone numbers. Subsequently, the research team sent the study link to participants via Telegram and WhatsApp. Only students who had experienced suicidal thoughts during the past week, as determined by the Beck Suicidal Ideation Scale, were included in the study.

Participants completed the Beck Depression Inventory (BDI-II), Tehran Multidimensional Perfectionism Scale (TMPS), Beck Hopelessness Scale (BHS), and Repetitive Negative Thinking Questionnaire (RNTQ). To ensure data confidentiality, all research instruments were designed using Google Forms and coded for anonymous data collection. Participants received the Google Forms link via WhatsApp and Telegram. Additionally, to uphold ethical principles, the informed consent form explicitly stated that, while all efforts were made to maintain confidentiality, the use of WhatsApp and Telegram platforms inherently posed some privacy considerations. To facilitate access to medical services, participants were given the option to contact the researcher via email if they wished to receive medical intervention or support. All the students participated voluntarily in the study and they were assured that their information would be confidential. The authors followed ethical considerations based on the Helsinki declaration (1975), as revised in 2008. In addition, this research has a code of ethics (IR.SBMU.RETECH.REC.1401.130) from the Research Vice-Chancellor of Shahid Beheshti University of Medical Sciences.

### Measures

#### Beck Scale for Suicidal Ideation (BSSI)

Designed by Beck et al. in 1997, this 19-item self-report scale includes three subscales of desire to die, readiness to commit suicide, and actual desire to commit suicide. The first five items consist of screening questions. This Likert type scale is rated from zero to two (24). The convergent validity of the Persian version of the Beck scale for suicidal ideation with SCL-90 showed 0.57 for depression and 0.46 for anxiety, and Cronbach’s alpha was reported to be 0.82 (25). In the present study, Cronbach’s alpha was found to be 0.91.

#### Beck Depression Inventory (BDI-II)

Beck Depression Inventory was developed by Beck et al. in 1996 and has 21 items. This inventory was designed to measure the severity of depressive symptoms in individuals over 13 years old (26) and each item is scored from zero to three. The inventory has been adapted in Iran and Cronbach’s alpha of the Iranian version was found to be 0.83. Additionally, the convergent validity of the inventory with Beck anxiety inventory and DASS scale were 0.45 and 0.55, respectively (27). In the present study, Cronbach’s alpha was found to be 0.90.

#### Tehran Multidimensional Perfectionism Scale (TMPS)

On the basis of Hewit and Flett’s multidimensional perfectionism scale, this scale was designed by Besharat in 2007. This 30- item scale consists of three dimensions of self-oriented, other-oriented, and socially-prescribed perfectionism. The scoring of this scale is based on a 5-point Likert type from 1 to 5. The content validity of this scale has been reported to be 0.80, 0.72, and 0.69 for self-oriented, other-oriented, and socially-prescribed perfectionism, respectively. The test-retest reliability of the scale was examined twice using a two to four-week interval and the results indicated a retest coefficient for self-oriented perfectionism, other-oriented perfectionism and socially prescribed perfectionism 0.85, 0.79, and 0.84 respectively (28). In the present study, Cronbach’s alpha was found to be 0.87, 0.77, and 0.89 for self-oriented, other-oriented, and socially-prescribed perfectionism, respectively.

#### Beck Hopelessness Scale (BHS)

Developed by Beck et al. in 1974, this is a 20-item scale which is answered as true or false. This three-dimensional scale measures hopelessness in 16-18 years old subjects. The severity classifications are: 2-3 = very low, 4-8 = mild, 9-14 = moderate, and 15-20= severe. The retest coefficient of this scale with a 6-week interval was reported to be 0.66 (29). The internal consistency of the Persian version of the Beck Hopelessness scale was 0.87 and valid tool for evaluating hopelessness among Iranian people (30). In the present study, the content validity of the scale was confirmed by 5 clinical psychology experts and the Kuder-Richardson coefficient of this scale was reported to be 0.85.

#### Negative Repetitive Thinking Questionnaire (RNTQ)

This questionnaire was developed by McEvoy et al. in 2010 and consists of 10 items which are scored on a 5-point Likert scale from 1 (Not at all true) to 5 (Very true) (31). The concurrent validity of the Persian version was reported as 0.79 for the anxiety scale and 0.78 for the Beck Depression Inventory. Additionally, test-retest reliability and Cronbach’s alpha were reported to be 0.76 and 0.91, respectively (32). In the present study, the Cronbach’s alpha was 0.90.

#### Statistical analysis

To investigate the relationships between perfectionism, feeling of hopelessness, repetitive negative thinking, and depressive symptoms, data were analyzed using Pearson’s correlation in SPSS 22 and Amos 24. Additionally, structural equation modeling with the Maximum Likelihood method was used to assess the mediating role of repetitive negative thinking and feeling of hopelessness in the link between perfectionism and depressive symptoms. To evaluate model fit, absolute fit indices were used to assess the discrepancy between observed variances and covariances and those predicted by the model. These indices included AGFI (>0.90), GFI (>0.90), and RMR (≤1). To examine the distance between the research model and the independence model, as well as its proximity to the saturated model, incremental fit indices were employed, including CFI (>0.90), IFI (>0.90), and NFI (>0.90). Furthermore, parsimonious fit indices, such as RMSEA (<0.08) and χ²/df (<0.05), were used to account for the trade-off between model complexity and goodness of fit. To enhance the accuracy of indirect effect estimation, in line with methodological recommendations, bootstrapping with 5000 resamples and a 95% confidence interval was applied. Furthermore, the normality of the data distribution was verified before conducting SEM. Model fit was comprehensively assessed using multiple goodness-of-fit indices, including Chi-square (χ²), normed chi-square (χ²/df), Goodness of Fit Index (GFI), Adjusted Goodness of Fit Index (AGFI), Incremental Fit Index (IFI), Comparative Fit Index (CFI), Root Mean Square Residual (RMR), Normed Fit Index (NFI), and Root Mean Square Error of Approximation (RMSEA). A bootstrap sample of 5000 resamples with a 95% significance level was used to estimate indirect effects and compute confidence intervals for fit indices.

### Findings

A total of 500 responses were collected. However, after applying the inclusion and exclusion criteria, data from 355 participants were retained for analysis. The participants’ ages had a mean of 22.57 and a standard deviation of 2.31. In terms of gender, 190 (53.5%) were female, and 165 (46.5%) were male. Regarding the education level, 16 students (4.5%) were in their first year, 31 (8.7%) in their second year, 51 (14.4%) in their third year, 114 (32.1%) in their fourth year, 101 (28.5%) in their fifth year, 32 (9%) in their sixth year, and 10 (2.8%) in their seventh year of the program. The mean GPA of participants was 16.88 ± 1.35. Three hundred and thirty-eight (95.2%) students were single, 14 (3.9%) were married, and 3 (0.8%) were in a white marriage. In terms of ethnicity, 194 students (54.6%) were Persian, 64 (18%) were Azeri, 22 (6.2%) were Kurd, 30 (8.5%) were Lur, and 45 (12.7%) were Gilak. The mean participants’ scores on the Beck Scale for Suicidal Ideation was 6.39 ± 6.41. In addition, based on the results of the independent t-test, no significant difference was found between the scores of the research variables, i.e., suicidal ideation, depressive symptoms, perfectionism, and feelings of hopelessness, and the gender of the participants at the 0.01 level. Descriptive characteristics and correlations between the research variables are shown in **Table 1**.

**Table 1:** Mean, standard deviation, and bivariate correlations between perfectionism, hopelessness, repetitive negative thinking and depressive symptoms.

Variable	1	2	3	4	5	6
1 Self-Oriented	1					
2 Other-Oriented	0.54**	1				
3 Socially Prescribed	0.54**	0.42**	1			
4 Hopelessness	0.29**	0.21**	0.34**	1		
5 Repetitive negative thinking	0.46**	0.35**	0.46**	0.47**	1	
6 Depressive Symptoms	0.43**	0.38**	0.45**	0.61**	0.65**	1
Mean	33.86	31.16	32.52	8.27	32.39	16.32
Std. Deviation	7.89	6.47	8.37	5.00	8.15	10.91
Skewness	-0.13	-0.01	0.06	0.26	-0.00	0.95
Kurtosis	-0.41	0.17	-0.64	-0.79	-0.62	1.04
T test (p) for sex	0.16 (0.35)	0.26 (0.79)	0.10 (0.91)	0.82 (0.35)	0.74 (0.45)	0.81 (0.41)

**P<0.01.

The results in **Table 1** show a significant positive correlation between the research variables (p < 0.01). **Table 1** presents the mean and standard deviation for the study variables: self-oriented perfectionism (M = 33.86, SD = 7.89), other-oriented perfectionism (M = 31.16, SD = 6.47), socially prescribed perfectionism (M = 32.52, SD = 8.37), feelings of hopelessness (M = 8.27, SD = 5.00), repetitive negative thinking (M = 32.39, SD = 8.15), and depressive symptoms (M = 16.32, SD = 10.91), respectively. Additionally, as Skewness and Kurtosis values are between -2 and +2, the data follow a normal distribution, confirming the assumption of normality (33).

According to **Table 2**, all three dimensions of perfectionism were separately able to predict depressive symptoms in medical students with suicidal ideation. To clarify further, self-oriented perfectionism with a regression slope of (β=0.20, p>0.001), other-oriented perfectionism with a regression slope of (β=0.16, p>0.005), and socially prescribed perfectionism with a regression slope of (β=0.27, p>0.001) were able to predict depressive symptoms (p>0.01). In addition, these three dimensions of perfectionism totally explained 0.26% of the variance in depressive symptoms. Moreover, as shown in **Table 2**, the predictive power of socially prescribed perfectionism (β = 0.27) was greater than those of self-oriented perfectionism (β = 0.20) and other-oriented perfectionism (β = 0.16). In the following, structural equation analysis is applied to examine the role of the mediators.

**Table 2:** Regression slopes of perfectionism dimensions on depressive symptoms.

Variable	B	SE	β	CR	P	R	R^2^	Adjusted R^2^
Self-Oriented	0.28	0.08	0.20	3.43	0.001	0.52	0.27	0.26
Other-Oriented	0.26	0.09	0.16	2.85	0.005			
Socially Prescribed	0.36	0.07	0.27	4.99	0.001			

**Figure 1:**
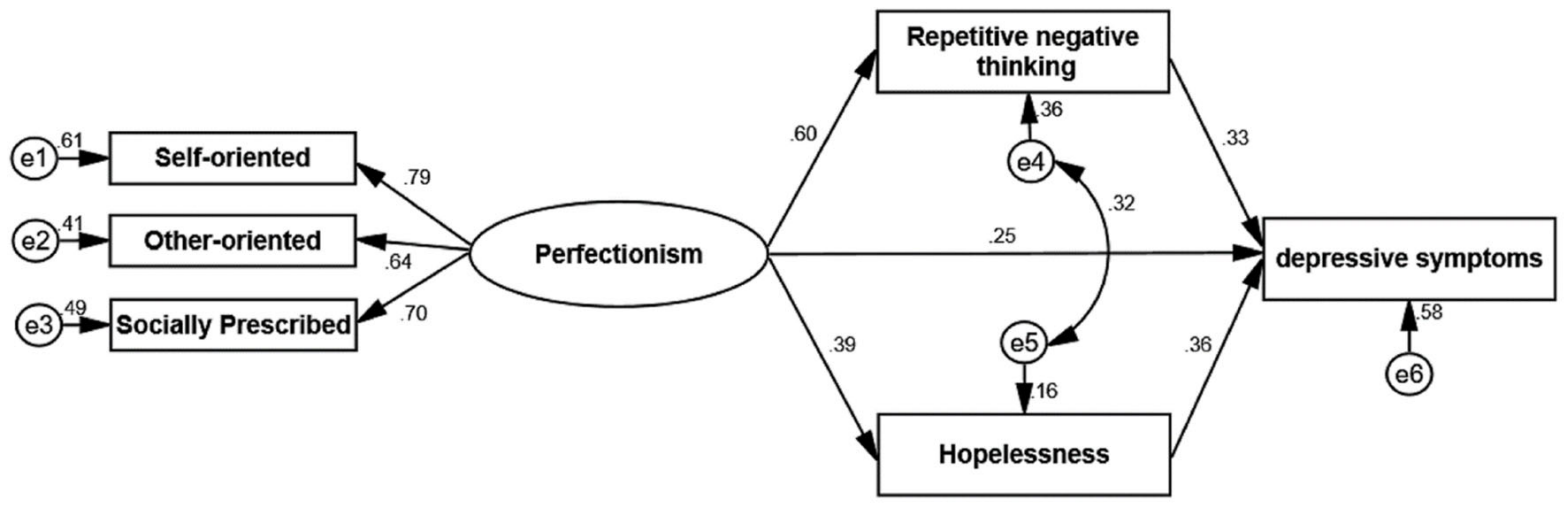
Regression coefficients of the mediation model of hopelessness and repetitive negative thinking in the relationship between perfectionism and depressive symptoms (*p* < 0.01).

**Figure 1** indicates the standard coefficients of the direct pathways of the hypothesized model for the mediating role of hopelessness and repetitive negative thinking in the relationship between perfectionism and depressive symptoms among medical students. Based on the results of structural equation modeling, the pathways between perfectionism and depressive symptoms with a regression slope of 0.25 (p < 0.001), perfectionism and hopelessness with a regression slope of 0.39 (p < 0.001), perfectionism and repetitive negative thinking with a regression slope of 0.60 (p < 0.001), hopelessness and depressive symptoms with a regression slope of 0.36 (p < 0.001), and repetitive negative thinking and depressive symptoms with a regression slope of 0.33 (p < 0.001) are all significant (p < 0.01).

Based on the results in **Table 3**, the direct pathways of perfectionism with hopelessness and repetitive negative thinking, with regression slopes of 0.39 (Lower = 0.28, Upper = 0.51, p < 0.001) and 0.60 (Lower = 0.48, Upper = 0.70, p < 0.001), respectively, as well as the direct pathways of hopelessness and repetitive negative thinking with depressive symptoms, with regression slopes of 0.36 (Lower = 0.28, Upper = 0.44, p < 0.001) and 0.33 (Lower = 0.22, Upper = 0.42, p < 0.001), respectively, were significant at the 0.01 level. Moreover, not only was perfectionism directly related to depressive symptoms with regression slopes of 0.25 (Lower = 0.14, Upper = 0.37, p < 0.001), but it also indirectly affected it through hopelessness (β = 0.35, Lower = 0.23, Upper = 0.47, p < 0.001) and repetitive negative thinking (β = 0.37, Lower = 0.26, Upper = 0.51, p < 0.001). The total indirect effect of perfectionism on depressive symptoms was 0.34. Accordingly, perfectionism both directly and indirectly plays a role in aggravating depressive symptoms among medical students by increasing feelings of hopelessness and repetitive negative thinking. In addition, the research findings showed that perfectionism, feelings of hopelessness, and repetitive negative thinking explain 58% of the variance in depressive symptoms (R² = 0.58). **Table 4** shows the results of model fit evaluation.

**Table 3:** Results of structural equation analysis for the general conceptual model and bootstrapping indirect effect and 95% confidence intervals.

direct and indirect pathways	β	SE	CR	p	Total effect	Lower	Upper
Perfectionism - Depressive Symptoms	0.25	0.10	4.36	0.001	–	0.14	0.37
Perfectionism - Hopelessness	0.39	0.05	6.35	0.001	–	0.28	0.51
Perfectionism - Repetitive negative thinking	0.60	0.09	9.43	0.001	–	0.48	0.70
Hopelessness - Depressive Symptoms	0.36	0.09	8.82	0.001	–	0.28	0.44
Repetitive negative thinking - Depressive Symptoms	0.33	0.07	6.64	0.001	–	0.22	0.42
Perfectionism - HOP - Depressive Symptoms	0.35	0.06	5.53	0.001	0.34	0.23	0.47
Perfectionism - RNT - Depressive Symptoms	0.37	0.06	6.16	0.001		0.26	0.51

**Table 4:** Goodness of fit indices for the mediation model of perfectionism, hopelessness, repetitive negative thinking and depressive symptoms.

GF Index	χ^2^	df	p	χ^2^/df	IFI	GFI	AGFI	CFI	NFI	RMR	RMSEA
Model	10.59	6	0.0	1.82	0.99	0.99	0.96	0.99	0.99	0.02	0.04
Threshold	–	–	> 0.05	< 5	> 0.90	> 0.90	> 0.90	> 0.90	> 0.90	≤ 1	< 0.08

According to **Table 4**, since the absolute fit indices, including the Goodness of Fit Index (GFI) = 0.99 and Adjusted Goodness of Fit Index (AGFI) = 0.96, are both greater than 0.90, it can be concluded that the research model has high goodness of fit. In addition, all comparative fit indices, including the Incremental Fit Index (IFI = 0.99), Comparative Fit Index (CFI = 0.99), and Normed Fit Index (NFI = 0.99), all exceed 0.90, indicating that the model fits well and closely approaches the saturated model. Furthermore, the parsimony fit indices, including Root Mean Square Residual (RMR), Normed Fit Index (NFI), Root Mean Square Error of Approximation (RMSEA), and the normed chi-square (χ²/df), fall within the acceptable range, suggesting a good model fit based on comparative, absolute, and parsimony fit indices, with empirical data supporting the model.

## Discussion

This study sought to provide an integrative model of depressive symptoms among medical students, investigating the role of RNT and feelings of hopelessness as potential links between perfectionism and depressive symptoms. Our results reinforce previous findings suggesting that perfectionism, RNT, and feelings of hopelessness are associated with an increased risk of depressive symptoms (34–36). Regarding perfectionism, our findings further support previous studies indicating that it increases the risk of developing depressive symptoms over time (9). Additionally, the significant relationship between RNT and depressive symptoms aligns with earlier research (36). This supports the transdiagnostic hypothesis, which posits that higher levels of RNT are linked to greater levels of depressive symptoms (37). Finally, studies supporting the hopelessness theory of depression can illuminate the association between feelings of hopelessness and depressive symptoms (35).

Additionally, current findings indicated that RNT serves as a mediator in the relationship between perfectionism and depressive symptoms. This result is consistent with prior studies that have identified various forms of RNT as mediators in the association between perfectionism and depressive symptoms (38–40). In line with these findings, previous studies have indicated that individuals with high levels of perfectionism are more likely to employ maladaptive coping strategies, such as RNT, instead of engaging in active problem-solving when faced with setbacks (41). Specifically, perfectionistic students are cognitively preoccupied with their past mistakes and future problems. This tendency leads them to focus on the ways in which they and their lives are not ideal (42). Consequently, they may experience depression due to their perception of a significant gap between their actual self (who they truly are) and their ought self (who they believe they should be) (43).

Moreover, our findings support the mediating role of feelings of hopelessness in the relationship between perfectionism and depressive symptoms in medical students experiencing SI. This aligns with previous findings that suggest feelings of hopelessness is a cognitive distortion arising from perfectionism (44, 45), which can contribute to depressive symptoms (35). According to PSDM, perfectionism is understood as a personality-related vulnerability, and self-criticism can arise as a possible reaction to the social disconnection often associated with perfectionistic tendencies (46). On the other hand, self-criticism may indicate that the self is permanently “bad” and will not get better (i.e., experiencing the feelings of hopelessness) (47). Furthermore, beyond the feelings of hopelessness when judging and criticizing the self, individuals with a history of mental health conditions are vulnerable to experience SI when self-critical (47). Taken together, the PSDM suggests that both the subjective and objective social disconnection experienced by perfectionists leads to feelings of low self-worth, self-criticism, and hopelessness. These feelings can ultimately result in negative consequences, such as depressive symptoms including suicidality (46, 47).

Generally, to extend previous studies, we developed an integrated model incorporating feelings of hopelessness and RNT to provide new insights into the development and intervention of depressive symptoms among perfectionistic medical students in Iran. This study suggests that medical students can reduce depression by changing their negative thinking. This change can help students develop positive coping strategies, thereby promoting mental health. Moreover, the study indicates that to reduce depression, medical students need to form personal skills to acquire reasonable expectations instead of self-criticism in response to hopeless situations.

### Limitations

Despite the strengths mentioned above, the findings should be interpreted in the context of its limitations. First, we utilized a purposive sampling method, which may skew the representation of medical students. For instance, participants who are more motivated might be overrepresented, potentially leading to biased result. To mitigate bias and the limitations associated with this method, future studies should consider employing random sampling methods. Second, self-reported inventories were utilized, which inherently involve subjective responses. This method may lead to underreporting of sensitive variables like SI due to social desirability bias. Consequently, these findings should be replicated using additional data collection methods, such as interviews or mixed-methods approaches, to provide a more comprehensive understanding of the phenomena. Third, the demographic variables of the participants were not controlled, which could influence the results. For example, differences in age, gender, or socioeconomic status might affect the relationship between perfectionism and depressive symptoms. Future studies should examine these variables to better understand their impact. Fourth, the absence of a mental disorder diagnosis was assessed through participants’ self-report statements. ​Utilizing the Structured Clinical Interview for DSM-5-ClinicianVersion (SCID-5-CV) would be a more accurate approach for future studies. Fifth, since the current study employed a cross-sectional design, we are unable to establish causation. The nature of the cause-and-effect relationship between the study variables will be better demonstrated by a longitudinal design, which can track changes over time and provide insights into the temporal relationships between perfectionism, RNT, feelings of hopelessness, and depressive symptoms. Sixth, the study’s findings indicate that participants exhibited mild levels of depression and hopelessness, which may suggest they experienced passive SI rather than active SI. This could limit the generalizability of the results to populations with more severe symptoms or active SI. To enhance the applicability of the findings, future studies should consider recruiting participants with a broader range of symptom severity. Future studies can also explore additional underlying variables in the association between perfectionism and depressive symptoms in medical students. Utilizing other variables (e.g., defeat, entrapment, and catastrophic thinking) to better understand the nature of depression among students experiencing SI is a valuable recommendation for future studies.

In terms of research sample and population, given that the study was conducted exclusively on medical students in Tehran, generalizing the findings to other cultural contexts and medical student populations should be done with caution. Additionally, as 95.2% of participants were single, caution is warranted in extrapolating the results to married students. Moreover, since more than half of the participants were Persian, findings should be interpreted cautiously when applied to students from different ethnic backgrounds. Furthermore, most participants were in academic years 3 to 7. Considering that medical education in Iran lasts seven years, with 3.5 years of preclinical theoretical training and 3.5 years of clinical practice, findings may not be fully generalizable to first- and second-year medical students.

### Practical implications

Our findings have significant implications for preventing and intervening in depressive symptoms among perfectionistic medical students in Iran. For instance, universities can mitigate the risk of depressive symptoms by establishing campus initiatives and offering open mental health courses for students. These programs can include workshops on stress management and cognitive-behavioral therapy tailored to address perfectionism, RNT, and feelings of hopelessness. Additionally, for professionals involved in psychological counseling with medical students, it is crucial to acknowledge the detrimental effects of perfectionism, RNT, and hopelessness. In light of these findings, policymakers can support universities by allocating resources for mental health services and promoting policies that reduce academic pressures. More specifically, policymakers should promote a balanced approach that emphasizes both academic success and mental health. Additionally, they should prioritize interventions addressing perfectionism via RNT and feelings of hopelessness to reduce depressive symptoms in future healthcare professionals.

## Conclusion

To our knowledge, the current study is the first to explore the chain mediating role of repetitive negative thinking (RNT) and feelings of hopelessness in the relationship between medical students’ perfectionism and depressive symptoms. Results suggest that perfectionism, RNT, and feelings of hopelessness are critical targets for interventions aimed at interrupting the sequence of perfectionism that ultimately leads to depressive symptoms. Furthermore, the findings of this study contribute to the existing literature by providing a deeper understanding of the underlying mechanisms linking these psychological factors. We hope that these insights can be utilized to intervene and identify risk factors at an early stage, thereby helping to minimize the risk of suicide among medical students.

## Data Availability

The raw data supporting the conclusions of this article will be made available by the authors, without undue reservation.

## References

[1] Harmer B Lee S Duong T v. H Saadabadi A. Suicidal Ideation. Treasure Island (FL: StatPearls Publishing (2023). Available at: http://europepmc.org/abstract/MED/33351435 (Accessed December 4, 2024). 33351435

[2] Coentre R Góis C. Suicidal ideation in medical students: recent insights. Adv Med Educ Pract. (2018) 9:873–80. doi: 10.2147/AMEP.S162626 PMC6276609 30568525

[3] Rotenstein LS Ramos MA Torre M Segal JB Peluso MJ Guille C. Prevalence of depression, depressive symptoms, and suicidal ideation among medical students: a systematic review and meta-analysis. Jama. (2016) 316:2214–36. doi: 10.1001/jama.2016.17324 PMC5613659 27923088

[4] Tyssen R Vaglum P Grønvold NT Ekeberg Ø. Suicidal ideation among medical students and young physicians: a nationwide and prospective study of prevalence and predictors. J Affect Disord. (2001) 64:69–79. doi: 10.1016/S0165-0327(00)00205-6 11292521

[5] Torres AR Campos LM Lima MCP Ramos-Cerqueira ATA. Suicidal ideation among medical students: prevalence and predictors. J Nervous Ment Dis. (2018) 206:160–8. doi: 10.1097/NMD.0000000000000734 28837427

[6] Moir F Yielder J Sanson J Chen Y. Depression in medical students: current insights. Adv Med Educ Pract. (2018) 9:323–33. doi: 10.2147/AMEP.S137384 PMC5944463 29765261

[7] Moutinho ILD Maddalena N d. CP Roland RK Lucchetti ALG Tibiriçá SHC. Depression, stress and anxiety in medical students: A cross-sectional comparison between students from different semesters. Rev Da Associação Med Bras. (2017) 63:21–8. doi: 10.1590/1806-9282.63.01.21 28225885

[8] Polujanski S Rotthoff T Nett U Schindler A-K. First-year medical students’ varying vulnerability to developing depressive symptoms and its predictors: a latent profile analysis. Acad Psychiatry. (2023) 47:143–51. doi: 10.1007/s40596-023-01757-x PMC9977089 36859506

[9] Smith MM Sherry SB Ray C Hewitt PL Flett GL. Is perfectionism a vulnerability factor for depressive symptoms, a complication of depressive symptoms, or both? A meta-analytic test of 67 longitudinal studies. Clin Psychol Rev. (2021) 84:101982. doi: 10.1016/j.cpr.2021.101982 33556805

[10] Hewitt PL Flett GL. Perfectionism in the self and social contexts: conceptualization, assessment, and association with psychopathology. J Pers Soc Psychol. (1991) 60:456. doi: 10.1037/0022-3514.60.3.456 2027080

[11] Etherson ME Smith MM Hill AP Sherry SB Curran T Flett GL. Perfectionism, mattering, depressive symptoms, and suicide ideation in students: A test of the Perfectionism Social Disconnection Model. Pers Individ Dif. (2022) 191:111559. doi: 10.1016/j.paid.2022.111559

[12] Mehr KE Adams AC. Self-compassion as a mediator of maladaptive perfectionism and depressive symptoms in college students. J Coll Student Psychother. (2016) 30:132–45. doi: 10.1080/87568225.2016.1140991

[13] Beck AT Alford BA. Depression: Causes and treatment. Philadelphia: University of Pennsylvania Press (2009).

[14] Klonsky ED May AM Saffer BY. Suicide, suicide attempts, and suicidal ideation. Annu Rev Clin Psychol. (2016) 12:307–30. doi: 10.1146/annurev-clinpsy-021815-093204 26772209

[15] Abramson LY Alloy LB Metalsky GI. Hopelessness depression. In: Explanatory style. London: Routledge (2014). p. 113–34.

[16] Marchetti I Loeys T Alloy LB Koster EH. Unveiling the structure of cognitive vulnerability for depression: Specificity and overlap. PloS One. (2016) 11:e0168612. doi: 10.1371/journal.pone.0168612 27992548 PMC5161451

[17] Békés V Dunkley DM Taylor G Zuroff DC Lewkowski M Foley JE. Chronic stress and attenuated improvement in depression over 1 year: The moderating role of perfectionism. Behav Ther. (2015) 46:478–92. doi: 10.1016/j.beth.2015.02.003 26163712

[18] Hewitt PL Norton GR Flett GL Callander L Cowan T. Dimensions of perfectionism, hopelessness, and attempted suicide in a sample of alcoholics. Suicide Life-Threatening Behav. (1998) 28:395–406. doi: 10.1111/j.1943-278X.1998.tb00975.x 9894307

[19] Smith MM Sherry SB McLarnon ME Flett GL Hewitt PL Saklofske DH. Why does socially prescribed perfectionism place people at risk for depression? A five-month, two-wave longitudinal study of the Perfectionism Social Disconnection Model. Pers Individ Dif. (2018) 134:49–54. doi: 10.1016/j.paid.2018.05.040

[20] Watkins ER. Constructive and unconstructive repetitive thought. psychol Bull. (2008) 134:163. doi: 10.1037/0033-2909.134.2.163 18298268 PMC2672052

[21] Papageorgiou C. “Worry and rumination: Styles of persistent negative thinking in, anxiety and depression. Worry and its psychological disorders: Theory, assessment and treatment”. In: Davey GCL Wells A, editor. Worry And Its Psychological Disorders: Theory, Assessment and Treatment. New Jersey: Wiley. (2006). p. 21–40. doi: 10.1002/9780470713143

[22] Flett GL Nepon T Hewitt PL. “Perfectionism, Worry, and Rumination in Health and Mental Health: A Review and a Conceptual Framework for a Cognitive Theory of Perfectionism”. In: Sirois F Molnar D, editor. Perfectionism, Health, and Well-Being. New York: Springer. (2016). p. 121–55. doi: 10.1007/978-3-319-18582-8_6

[23] Kline T. Classical test theory: Assumptions, equations, limitations, and item analyses. psychol Testing: A Pract Approach To Design Eval. (2005) 91:77–90. doi: 10.4135/9781483385693

[24] Beck AT Steer RA Ranieri WF. Scale for suicide ideation: Psychometric properties of a self-report version. J Clin Psychol. (1988) 44:499–505. doi: 10.1002/1097-4679(198807)44:4<499::AID-JCLP2270440404>3.0.CO;2-6 3170753

[25] Esfahani M Hashemi Y Alavi K. Psychometric assessment of beck scale for suicidal ideation (BSSI) in general population in Tehran. Med J Islamic Republic Iran. (2015) 29:268. PMC4715388 26793659

[26] Beck AT Steer RA Brown GK. Beck depression inventory. New York: Springer (1996).

[27] Toosi F Rahimi C Sajjadi S. Psychometric properties of beck depression inventory-II for high school children in Shiraz City, Iran. Int J School Health. (2017) 4:1–6. doi: 10.5812/intjsh.41069

[28] Besharat MA. Development and validation of the tehran multidimensional perfectionism scale. psychol Res. (2007) 10:49–67.

[29] Beck AT Weissman A Lester D Trexler L. The measurement of pessimism: the hopelessness scale. J Consulting Clin Psychol. (1974) 42:861. doi: 10.1037/h0037562 4436473

[30] Sadeghian MH Etesam F Nakhostin-Ansari A Akbarpour S Akhlaghi M. Association between hopelessness and suicidal ideation in Iranian medical students: a cross-sectional study. Health Psychol Res. (2021) 9:27579. doi: 10.52965/001c.27579 35106399 PMC8801550

[31] McEvoy PM Thibodeau MA Asmundson GJ. Trait repetitive negative thinking: A brief transdiagnostic assessment. J Exp Psychopathol. (2014) 5:1–17. doi: 10.5127/jep.037813

[32] Akbari M. Psychometric properties of repetitive thinking questionnaire in nonclinical sample: Trans diagnostic tool. J Clin Psychol. (2017) 9:59–72. doi: 10.22075/jcp.2017.10345

[33] Sarstedt M Ringle CM Hair JF. Partial least squares structural equation modeling. In: Handbook of market research. New York: Springer (2021). p. 587–632.

[34] Lea K Richardson T Rauze N. The relationship between mood symptom severity and perfectionism subtypes in mood disorders: A systematic review and meta-analysis. Brain Sci. (2023) 13:377. doi: 10.3390/brainsci13030377 36979187 PMC10045978

[35] Liu RT Kleiman EM Nestor BA Cheek SM. The hopelessness theory of depression: A quarter-century in review. Clin Psychol: Sci Pract. (2015) 22:345. doi: 10.1111/cpsp.12125 PMC4689589 26709338

[36] Spinhoven P van Hemert AM Penninx BW. Repetitive negative thinking as a predictor of depression and anxiety: A longitudinal cohort study. J Affect Disord. (2018) 241:216–25. doi: 10.1016/j.jad.2018.08.037 30138805

[37] Wahl K Ehring T Kley H Lieb R Meyer A Kordon A. Is repetitive negative thinking a transdiagnostic process? A comparison of key processes of RNT in depression, generalized anxiety disorder, obsessive-compulsive disorder, and community controls. J Behav Ther Exp Psychiatry. (2019) 64:45–53. doi: 10.1016/j.jbtep.2019.02.006 30851652

[38] Di Schiena R Luminet O Philippot P Douilliez C. Adaptive and maladaptive perfectionism in depression: Preliminary evidence on the role of adaptive and maladaptive rumination. Pers Individ Dif. (2012) 53:774–8. doi: 10.1016/j.paid.2012.05.017

[39] Macedo A Soares M Amaral A Nogueira V Madeira N Roque C. Repetitive negative thinking mediates the association between perfectionism and psychological distress. Pers Individ Dif. (2015) 72:220–4. doi: 10.1016/j.paid.2014.08.024

[40] O’Connor DB O’Connor RC Marshall R. Perfectionism and psychological distress: Evidence of the mediating effects of rumination. Eur J Personality: Published Eur Assoc Pers Psychol. (2007) 21:429–52. doi: 10.1002/per.616

[41] Park H-j Paul Heppner P Lee D-g. Maladaptive coping and self-esteem as mediators between perfectionism and psychological distress. Pers Individ Dif. (2010) 48:469–74. doi: 10.1016/j.paid.2009.11.024

[42] Garratt-Reed D Howell J Hayes L Boyes M. Is perfectionism associated with academic burnout through repetitive negative thinking? PeerJ. (2018) 6:e5004. doi: 10.7717/peerj.5004 29938132 PMC6011823

[43] Enns MW Cox BJ Clara I. Adaptive and maladaptive perfectionism: Developmental origins and association with depression proneness. Pers Individ Dif. (2002) 33:921–35. doi: 10.1016/S0191-8869(01)00202-1

[44] O’Connor RC O’Connor DB. Predicting hopelessness and psychological distress: The role of perfectionism and coping. J Couns Psychol. (2003) 50:362. doi: 10.1037/0022-0167.50.3.362

[45] Robinson A Moscardini E Tucker R Calamia M. Perfectionistic self-presentation, socially prescribed perfectionism, self-oriented perfectionism, interpersonal hopelessness, and suicidal ideation in US adults: Reexamining the social disconnection model. Arch Suicide Res. (2022) 26:1447–61. doi: 10.1080/13811118.2021.1922108 34019781

[46] Hewitt PL Flett GL Mikail SF Kealy D Zhang LC. Perfectionism in the therapeutic context: The perfectionism social disconnection model. In: The psychology of perfectionism. London: Routledge (2017). p. 306–30.

[47] Brott KH Veilleux JC. Examining state self-criticism and self-efficacy as factors underlying hopelessness and suicidal ideation. Suicide Life-Threatening Behav. (2024) 54:207–20. doi: 10.1111/sltb.13034 38112324

